# Inhibition of Bacterial Quorum Sensing by Extracts from Aquatic Fungi: First Report from Marine Endophytes

**DOI:** 10.3390/md12115503

**Published:** 2014-11-19

**Authors:** Alberto J. Martín-Rodríguez, Fernando Reyes, Jesús Martín, Juan Pérez-Yépez, Milagros León-Barrios, Alan Couttolenc, César Espinoza, Ángel Trigos, Víctor S. Martín, Manuel Norte, José J. Fernández

**Affiliations:** 1 Institute for Bioorganic Chemistry “Antonio González”, Center for Biomedical Research of the Canary Islands (CIBICAN), University of La Laguna, Av. Astrofísico Francisco Sánchez 2, 38206 La Laguna, Tenerife, Spain; E-Mails: vmartin@ull.es (V.S.M.); mnorte@ull.es (M.N.); 2 Oceanic Platform of the Canary Islands (PLOCAN), Carretera de Taliarte s/n, 35214 Telde, Gran Canaria, Spain; 3 Fundación MEDINA, Parque Tecnológico de Ciencias de la Salud, Av. del Conocimiento 34, 18016 Armilla, Granada, Spain; E-Mails: fernando.reyes@medinaandalucia.es (F.R.); jesus.martin@medinaandalucia.es (J.M.); 4 Department of Microbiology, Biochemistry, Cellular Biology and Genetics, Faculty of Health Science, University of La Laguna, Av. Astrofísico Francisco Sánchez s/n, 38071 La Laguna, Tenerife, Spain; E-Mails: juanernestoperezyepez@gmail.com (J.P.-Y.); mileonba@ull.es (M.L.-B.); 5 Ph.D. Program in Biomedical Sciences, Veracruzana University, Luis Castelazo Ayala s/n, Colonia Industrial Ánimas, 91190 Xalapa, Veracruz, Mexico; E-Mail: a_cuto11@hotmail.com; 6 Xalapa High Technology Laboratory (LATEX), Veracruzana University, Médicos 5, Unidad del Bosque, 91010 Xalapa, Veracruz, Mexico; E-Mails: cespinoza@uv.mx (C.E.); atrigos@uv.mx (A.T.)

**Keywords:** quorum sensing, fungi, LC-HRMS, metabolic profile, endophytes, *Chromobacterium violaceum*, biofouling, antifouling

## Abstract

In our search for quorum-sensing (QS) disrupting molecules, 75 fungal isolates were recovered from reef organisms (endophytes), saline lakes and mangrove rhizosphere. Their QS inhibitory activity was evaluated in *Chromobacterium violaceum* CVO26. Four strains of endophytic fungi stood out for their potent activity at concentrations from 500 to 50 μg mL^−1^. The molecular characterization, based on the internal transcribed spacer (ITS) region sequences (ITS1, 5.8S and ITS2) between the rRNA of 18S and 28S, identified these strains as belonging to four genera: *Sarocladium* (LAEE06), *Fusarium* (LAEE13), *Epicoccum* (LAEE14), and *Khuskia* (LAEE21). Interestingly, three came from coral species and two of them came from the same organism, the coral *Diploria strigosa*. Metabolic profiles obtained by Liquid Chromatography-High Resolution Mass Spectrometry (LC-HRMS) suggest that a combination of fungal secondary metabolites and fatty acids could be the responsible for the observed activities. The LC-HRMS analysis also revealed the presence of potentially new secondary metabolites. This is, to the best of our knowledge, the first report of QS inhibition by marine endophytic fungi.

## 1. Introduction

Quorum sensing (QS) is the term coined to describe the process of cell-to-cell communication in bacteria. This intercellular communication enables the execution of coordinated behaviors in function of the bacterial population density. The process relies on the production, release and reception of signaling molecules that have been often classified into three main chemical categories: *N*-acyl homoserine lactones (AHLs), also referred as “autoinducer-1” (AI-1), which are characteristic of Gram-negative bacteria; autoinducing peptides (AIPs), employed by Gram-positive bacteria; and the so-called “autoinducer-2” (AI-2), furanones whose common precursor is (*S*)-4,5-dihydroxy-2,3-pentanedione (DPD) that have been proposed as a universal signal between Gram-positive and Gram-negative bacteria [1]. Nevertheless, other chemically different signaling molecules have also been identified over the last decades, such as the A-factor from *Streptomyces* (a γ-butyrolactone), 4-quinolones (*Pseudomonas* Quinolone Signal, PQS) or fatty acids (Diffusible Signal Factors, DSF) [2,3]. The most recent example are pyrones, recently identified as QS signals in *Photorhabdus luminescens* [4,5]. This likely represents only a small proportion of the extracellular metabolites involved in QS signaling. Similarly, the chemical nature of the molecules that are able to agonize or antagonize this phenomenon is equally diverse [6].

Once a threshold concentration of autoinducers is achieved in the extracellular milieu, bacterial gene expression is altered. These QS-regulated genes are involved in a variety of processes: production of virulence factors and secondary metabolites, sporulation, competence, or biofilm formation, among others [7]. For this reason, inhibition of QS constitutes a key target in the control of biofilm-related problems, including marine biofouling. Biofouling, the undesirable settlement of marine organisms on immersed substrata, begins with the adsorption of organic matter and the formation of bacterial biofilms, which in turn modulate the settlement of macroscopic foulers [8,9]. For example, *Ulva* zoospores are able to recognize AHLs from bacterial biofilms as a chemoattractive cue for settlement [10,11]. Spore release in the epiphytic alga *Acrochaetium* is also induced by bacterial AHLs [12]. Recently, a direct correlation between AHL concentration and cyprid settlement has been found in *Balanus improvises* [11]. As (ideally) QS blockers do not target bacterial growth, they do not exert a selective pressure on bacterial populations. In fact, this strategy is widely employed by nature to interfere with bacterial colonization, either by mimicking the bacterial autoinducers (e.g., brominated furanones from *Delisea pulchra*) [13,14], or by “quenching” bacterial signals through enzymatic degradation (e.g., lactonases from *Bacillus*, *Pseudomonas* or *Shewanella* species) [15].

Fungi are a renowned source of products with an array of bioactivities, from antibacterial to antiviral, cytotoxic, antiinflammatory, antifeeding, antifungal or antioxidant, among many others [16,17,18]. In recent years, research on fungi associated with marine invertebrates and algae has revealed the presence of antifouling secondary metabolites [19,20]. Indeed, there is increasing evidence that many of the bioactive metabolites produced by sponges or algae as chemical defenses to avoid epibiosis are not truly produced by these organisms themselves, but by microbes—mainly bacteria and fungi—living in association with them [21,22]. Although bacterial-fungal interactions are largely documented [23,24,25], there are relatively few reports on QS inhibition by fungal metabolites. For instance, Rasmussen and co-workers identified patulin and penicillic acid from *Penicillium* species as QS inhibitors (QSIs) in *P*. *aeruginosa* [26]. Conversely, the fungal QS molecule farnesol from *Candida albicans* has been reported to inhibit the production of PQS in *P. aeruginosa*, which in turn prevents the yeast from entering in the filamentous phase through the production of the QS inducer 3-oxo-C12 homoserine lactone [27,28].

As part of our search for novel antifouling agents, a collection of 75 strains of fungi were isolated from aquatic habitats in the states of Veracruz and Puebla, Mexico, cultured at laboratory scale and screened for the presence of QSIs with the reporter strain *Chromobacterium violaceum* CVO26. QSI production was screened in extracts from both the fungal biomass and its culture medium. For the most active isolates, a phylogenetic analysis by amplification of the ITS region (ITS1, 5.8S and ITS2) was conducted to assess a genetic identification. In order to investigate the chemical nature of the fungal metabolites involved in the observed bioactivity, LC-HRMS profiles of the most active extracts were analyzed.

## 2. Results

### 2.1. Sampling and Isolation

**Figure 1:**
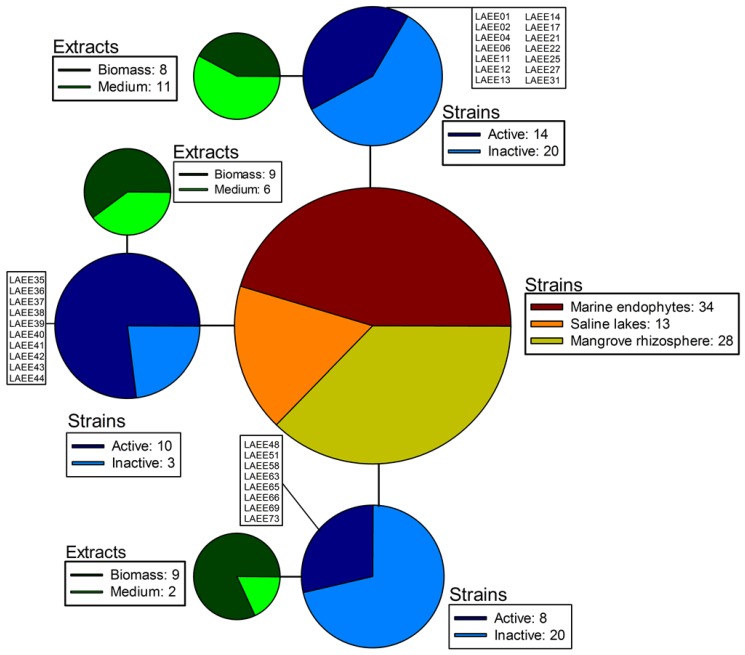
Distribution of the strains isolated in this study in function of their origin (central circle); proportion of active and inactive strains in each case (blue circles); and origin (biomass or culture medium) of the active extracts in each case (green circles).

Three different ecological niches were selected for the isolation of fungal strains: endophytes from reef organisms (corals, sponges, and algae), mangrove rhizosphere soils, and saline lakes. These are all aquatic ecosystems with marked differences that are likely to host a rich microbial diversity. Indeed, the sampling resulted in 75 isolates belonging to 21 genera (Table 1). The marine sources were particularly prolific, since more than half the isolates (34) were endophytes, followed by those associated with mangrove roots (28). Altogether, these two groups accounted for 83% of the isolates (Figure 1).

**Table 1:** Strains of fungi isolated from Mexican aquatic habitats.

Strain ID.	Fungus Genus	Biological Source	Location
LAEE 01	*Cladosporium* sp.	*Amphimedon compressa*	Arrecifes Blancas
LAEE 02	*Aspergillus* sp.	*Amphimedon compressa*	Arrecifes Blancas
LAEE 03	*Curvularia* sp.	*Amphimedon compressa*	Arrecifes Blancas
LAEE 04	*Fusarium* sp.	*Agelas* sp.	Arrecifes Blancas
LAEE 05	*Aspergillus* sp.	*Agelas* sp.	Arrecifes Blancas
LAEE 06	*Sarocladium* sp.	*Agelas* sp.	Arrecifes Blancas
LAEE 07	*Fusarium* sp.	*Aplysina* sp.	Arrecifes Blancas
LAEE 08	*Acremonium* sp.	*Chondrilla* sp.	Arrecifes Blancas
LAEE 09	*Fusarium* sp.	*Acropora palmata*	Arrecifes Blancas
LAEE 10	*Acremonium* sp.	*Acropora palmata*	Arrecifes Blancas
LAEE 11	*Acremonium* sp.	*Acropora palmata*	Arrecifes Blancas
LAEE 12	*Fusarium* sp.	*Diploria clivosa*	Arrecifes Blancas
LAEE 13	*Fusarium* sp.	*Diploria strigosa*	Arrecifes Blancas
LAEE 14	*Epicoccum* sp.	*Diploria strigosa*	Arrecifes Blancas
LAEE 15	*Trichoderma* sp.	*Diploria strigosa*	Arrecifes Blancas
LAEE 16	*Aspergillus* sp.	*Diploria strigosa*	Arrecifes Blancas
LAEE 17	*Fusarium* sp.	*Diploria strigosa*	Arrecifes Blancas
LAEE 18	*Aspergillus* sp.	*Montastrea cavernosa*	Arrecifes Blancas
LAEE 19	*Aspergillus* sp.	*Montastrea cavernosa*	Arrecifes Blancas
LAEE 20	*Aspergillus* sp.	*Montastrea cavernosa*	Arrecifes Blancas
LAEE 21	*Khuskia* sp.	*Plexaura flexuosa*	Arrecifes Blancas
LAEE 22	*Cladosporium* sp.	*Plexaura flexuosa*	Arrecifes Blancas
LAEE 23	*Trichoderma* sp.	*Plexaura flexuosa*	Arrecifes Blancas
LAEE 24	*Monilia* sp.	*Plexaura flexuosa*	Arrecifes Blancas
LAEE 25	*Acremonium* sp.	*Pseudoplexaura porosa*	Arrecifes Blancas
LAEE 26	*Acremonium* sp.	*Pseudoterogorgia americana*	Isla de Sacrificios
LAEE 27	*Aspergillus* sp.	*Pseudoterogorgia americana*	Isla de Sacrificios
LAEE 28	*Alternaria* sp.	*Siderastrea siderea*	Arrecifes Blancas
LAEE 29	*Aspergillus* sp.	*Siderastrea siderea*	Arrecifes Blancas
LAEE 30	*Aspergillus* sp.	*Siderastrea siderea*	Arrecifes Blancas
LAEE 31	*Fusarium* sp.	*Siderastrea siderea*	Arrecifes Blancas
LAEE 32	*Aspergillus* sp.	*Siderastrea siderea*	Arrecifes Blancas
LAEE 33	*Fusarium* sp.	*Zoanthus* sp.	Isla de Sacrificios
LAEE 34	*Aspergillus* sp.	*Hypnea cervicornis*	Isla de Sacrificios
LAEE 35	*Monilia* sp.	Sample from littoral zone	Laguna de Atexcac
LAEE 36	*Ulocladium* sp.	Sample from littoral zone	Laguna de Atexcac
LAEE 37	*Stachybotrys* sp.	Sample from littoral zone	Laguna de Atexcac
LAEE 38	*Fusarium* sp.	Sample from littoral zone	Laguna de Atexcac
LAEE 39	*Fusarium* sp.	Sample from littoral zone	Laguna de Atexcac
LAEE 40	*Absidia* sp.	*Potamogeton* sp.	Laguna de Atexcac
LAEE 41	*Paecilomyces* sp.	Sample from littoral zone	Laguna de Atexcac
LAEE 42	*Chaetomium* sp.	Sample from littoral zone	Laguna de Atexcac
LAEE 43	*Stemphylium* sp.	*Juncus* sp.	Laguna de Atexcac
LAEE 44	*Penicillium* sp.	*Cladophora* sp.	Laguna de Atexcac
LAEE 45	*Acremonium* sp.	Sample from littoral zone	Laguna de Atexcac
LAEE 46	*Aspergillus* sp.	Sample from littoral zone	Laguna de Atexcac
LAEE 47	*Aspergillus* sp.	*Cladophora* sp.	Laguna de Atexcac
LAEE 48	*Fusarium* sp.	Rhizosphere of *Rhizophora mangle*	Manglar de Tuxpan
LAEE 49	*Alternaria* sp.	Rhizosphere of *Rhizophora mangle*	Manglar de Tuxpan
LAEE 50	*Fusarium* sp.	Rhizosphere of *Rhizophora mangle*	Manglar de Tuxpan
LAEE 51	*Trichocladium* sp.	Rhizosphereof * Rhizophora mangle*	Manglar de Tuxpan
LAEE 52	*Aspergillus* sp.	Rhizosphere of *Rhizophora mangle*	Manglar de Tuxpan
LAEE 53	*Penicillium* sp.	Rhizosphere of *Rhizophora mangle*	Manglar de Tuxpan
LAEE 54	*Aspergillus* sp.	Rhizosphere of *Rhizophora mangle*	Manglar de Tuxpan
LAEE 55	*Fusarium* sp.	Rhizosphere of *Rhizophora mangle*	Manglar de Tuxpan
LAEE 56	*Aspergillus* sp.	Rhizosphere of *Rhizophora mangle*	Manglar de Tuxpan
LAEE 57	*Mucor* sp.	Rhizosphere of *Rhizophora mangle*	Manglar de Tuxpan
LAEE 58	*Cladosporium* sp.	Rhizosphere of *Rhizophora mangle*	Manglar de Tuxpan
LAEE 59	*Fusarium* sp.	Rhizosphere of *Rhizophora mangle*	Manglar de Tuxpan
LAEE 60	*Aspergillus* sp.	Rhizosphere of *Avicennia germinans*	Manglar de Tuxpan
LAEE 61	*Blastomyces* sp.	Rhizosphere of *Avicennia germinans*	Manglar de Tuxpan
LAEE 62	*Aspergillus* sp.	Rhizosphere of *Avicennia germinans*	Manglar de Tuxpan
LAEE 63	*Fusarium* sp.	Rhizosphere of *Avicennia germinans*	Manglar de Tuxpan
LAEE 64	*Fusarium* sp.	Rhizosphere of *Avicennia germinans*	Manglar de Tuxpan
LAEE 65	*Penicillium* sp.	Rhizosphere of *Avicennia germinans*	Manglar de Tuxpan
LAEE 66	*Acremonium* sp.	Rhizosphere of *Avicennia germinans*	Manglar de Tuxpan
LAEE 67	*Aspergillus* sp.	Rhizosphere of *Laguncularia racemosa*	Manglar de Tuxpan
LAEE 68	*Aspergillus* sp.	Rhizosphere of *Laguncularia racemosa*	Manglar de Tuxpan
LAEE 69	*Penicillium* sp.	Rhizosphere of *Laguncularia racemosa*	Manglar de Tuxpan
LAEE 70	*Acremonium* sp.	Rhizosphere of *Laguncularia racemosa*	Manglar de Tuxpan
LAEE 71	*Aspergillus* sp.	Rhizosphere of *Laguncularia racemosa*	Manglar de Tuxpan
LAEE 72	*Aspergillus* sp.	Rhizosphere of *Laguncularia racemosa*	Manglar de Tuxpan
LAEE 73	*Fusarium* sp.	Rhizosphere of *Laguncularia racemosa*	Manglar de Tuxpan
LAEE 74	*Paecilomyces* sp.	Rhizosphere of *Laguncularia racemosa*	Manglar de Tuxpan
LAEE 75	*Paecilomyces* sp.	Rhizosphere of *Laguncularia racemosa*	Manglar de Tuxpan

### 2.2. Quorum Sensing Assays

Extracts from the fungal biomasses and their liquid culture media were screened for the presence of QSIs with the reporter strain *C*. *violaceum* CVO26. Thus, two extracts (biomass and spent medium) were prepared for each fungal isolate. Two screening rounds were conducted. In the first-round screening, extracts were evaluated at 100 μg·mL^−1^. Extracts inhibiting violacein production above 40% with respect to the control were considered active samples. 14 of the 34 marine endophytic strains, 8 of the 28 mangrove root-associated fungi and 10 of the 13 saline lake-derived isolates exhibited activity, either from their broth extracts or their biomasses (Figure 1). 26 of these active samples (58%) belonged to fungal biomasses, suggesting that most of the bioactive molecules were not delivered to the extracellular milieu under the laboratory culture conditions.

**Figure 2:**
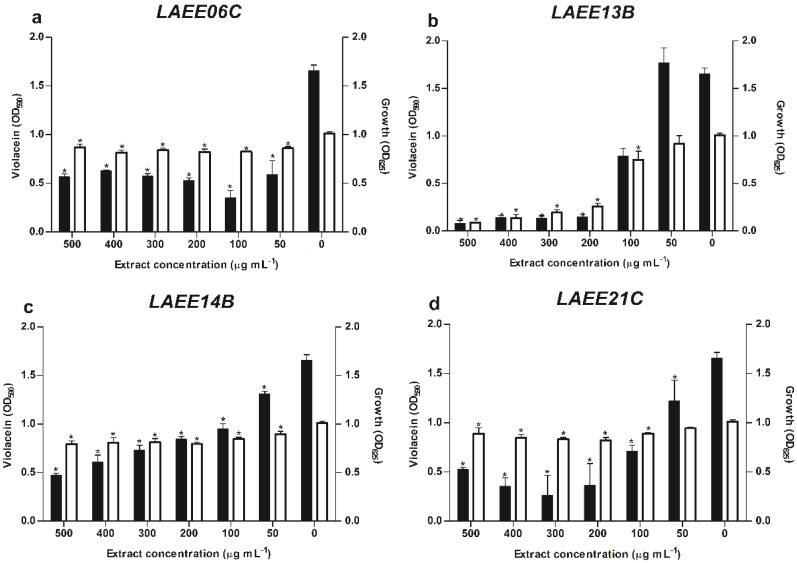
Growth inhibition (white bars) and violacein inhibition (black bars) caused by different concentration of the fungal extracts: (**a**) *LAEE06C*; (**b**) *LAEE13B*; (**c**) *LAEE14B* and (**d**) *LAEE21C*. Data represent the mean ± SD. Asterisks indicate significant differences respect the untreated control (one-way ANOVA, Dunnett’s multiple comparison test, *p* < 0.05).

The active samples were subjected to a second-round screening in which serial dilutions from 500 to 50 μg·mL^−1^ were evaluated (Figure 2). Four samples stood out for their high activity (inhibitions above 70% in violacein production): *LAEE06C* (strain LAEE06, culture medium extract), *LAEE13B* (strain LAEE13, biomass extract), *LAEE14B* (strain LAEE14, biomass extract) and *LAEE21C* (strain LAEE21, culture medium extract). These samples inhibited violacein production dose-dependently (Figure 2), all belonging to marine endophytic fungi. Interestingly, two of these isolates, LAEE13 and LAEE17, were isolated from the same coral species, *Diploria strigosa* (Table 1). Although statistically significant differences were quantified for bacterial growth in the presence of the fungal extracts (One-way ANOVA, Dunnett’s post-hoc test, *p* < 0.05), a dose-dependent trend on violacein production was observed (Figure 2). As evidenced in Figure 2a,c,d, this dose-dependent inhibition of violacein synthesis is not correlated with higher toxicities (*i.e*., growth inhibition) on the reporter strain. In effect, extracts *LAEE06C*, *LAEE14B* and *LAEE21C* seem to exert a basal effect on bacterial growth that does not change significantly in the range of tested concentrations. This is in marked contrast with the effect observed on violacein production, which is clearly inhibited by increasing concentrations of the fungal extracts. By contrast, extract *LAEE13B* exerted an evident antibacterial activity that explains by itself the dose-dependent phenotypic evidence (Figure 2b).

### 2.3. ITS Phylogeny and Taxonomic Identification of the Isolates

**Figure 3:**
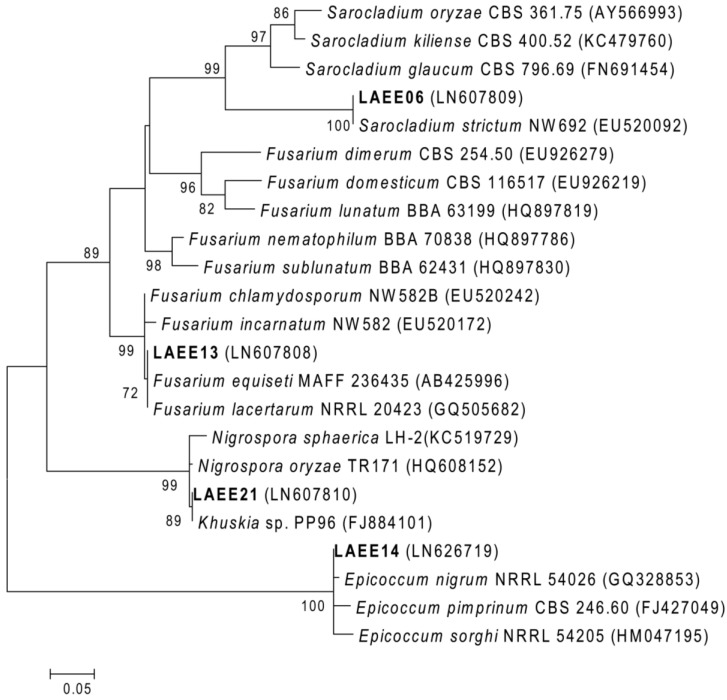
Maximum-likelihood (General Time Reversible, with gamma distribution) phylogenetic tree based on ITS sequences (552 nt) of isolates of this study and reference strains. Accession numbers are given in parentheses. Numbers at the nodes are bootstrap support values for 500 replicates. The scale bar indicates the number of substitutions per site.

ML and NJ tree reconstructions were used in the phylogenetic analysis, however, as both showed the same topology, only the ML tree is included in the text (Figure 3, Supplementary Figure S1). The four isolates were placed, with high statistical support (99% and 100% bootstrap values), on four different branches corresponding to the genera *Sarocladium* (LAEE06), *Fusarium* (LAEE13), *Epicoccum* (LAEE14), and *Khuskia* (LAEE21).

The isolate LAEE06, from genus *Sarocladium*, shared 99.8% sequence similarity with the species *S. strictum*, thus, this isolate could be considered a new strain of this species. Within the *Fusarium* cluster, LAEE13 occupied an intermediate position between two closely related lineages, the *F. incarnantum*-*F. equiseti* species complex (FIESC) and the *F. chlamydosporum* species complex (FCSC). So, with the current data, isolate LAEE13 cannot be assigned to a specific species, as the ITS sequences were not discriminative enough to resolve at the species level for this clade [29]. The closest relative of isolate LAEE14 is the species *Epicoccum nigrum*, with which it shares 99.4% sequence similarity, and it could be a new strain of *E. nigrum* or a novel sister species. In the *Khuskia*/*Nigrospora* cluster, *Nigrospora* (anamorph of *Khuskia*) *sphaerica* was the closest species of isolate LAEE21, with 98.8% sequence similarity. The ITS sequence of LAEE21, interestingly, was almost identical (99.8% similar) to another unclassified endophytic fungal isolate.

### 2.4. Metabolic Profiles

**Figure 4:**
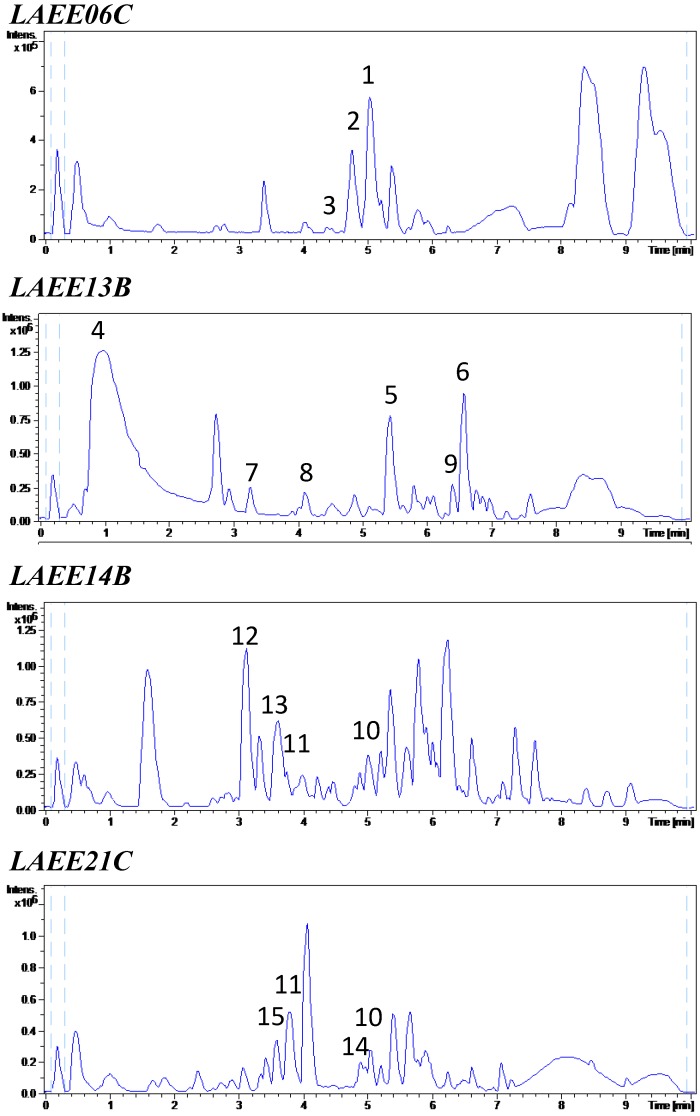
Electrospray ionization positive mode base peak chromatograms of the QS active samples. The numbers above the peaks identify the metabolites listed in Table 2.

In order to investigate the chemical nature of the observed inhibitions, LC-HRMS profiles of the four samples, *LAEE06C*, *LAEE13B*, *LAEE14B* and *LAEE21C*, were obtained. The identified metabolites are summarized in Table 2 and the most relevant chemical structures are included in Figure 4.

**Table 2:** Annotated peaks observed in the chromatograms of the extracts *LAEE06C*, *LAEE13B*, *LAEE14B* and *LAEE21C*.

Sample	RT (min)	Suggested MF	FM Database ID	Fungal Metabolites with This MF Included in the DNP
*LAEE06C*	4.36	C_27_H_49_O_12_P		Lysofungin (**3**)
	4.76	C_33_H_43_NO_4_		Antibiotic GKK 1032A_1_ (**2**)
	5.04	C_33_H_45_NO_4_		Sespendole (**1**)
	5.39	C_18_H_30_O_2_		Linolenic acid
	6.64	C_18_H_35_NO_2_		Not found in the DNP
*LAEE13B*	0.90	C_10_H_13_NO_2_	Fusaric acid (**4**)	
	2.74	C_9_H_13_NO		Not found in the DNP
	3.26	C_16_H_21_NO_4_		*N*-Acetyl-*O*-prenyltyrosine (**7**)
	4.02	C_13_H_16_O_3_		5 coincidences in the DNP
	4.11	C_18_H_30_O_3_		11-Oxo-9,12-octadecadienoic acid (**8**)
	5.41	C_21_H_29_NO_4_	Trichosetin (**5**)	
	6.39	C_44_H_55_N_3_O_9_		Beauvericin D (**9**)
	6.56	C_45_H_57_N_3_O_9_	Beauvericin (**6**)	
*LAEE14B*	3.29	C_20_H_22_N_4_O_2_		Phenylahistin (**12**)
	3.48	C_24_H_29_N_3_O_4_		Variecolorin N (**13**)
	3.76	C_23_H_32_O_7_		(*E*,*E*)-6-(6′,7′-dihydroxy-2′,4′-octadienoyl)-strobilactone A
	5.00	C_31_H_55_N_5_O_7_		Emericellamide A (**10**)
	5.74	C_15_H_20_O_3_		>50 coincidences in the DNP
	6.15	C_25_H_34_O		Not found in the DNP
	6.53	C_18_H_32_O_2_		Linoleic acid
*LAEE21C*	2.38	C_10_H_16_O_4_		13 coincidences in the DNP
	3.08	C_15_H_22_O_4_		>50 coincidences in the DNP
	3.59	C_18_H_34_O_5_		Penicitide B (**15**)
	3.79	C_23_H_32_O_7_		(*E*,*E*)-6-(6′,7′-dihydroxy-2′,4′-octadienoyl)-strobilactone A
	4.06	C_17_H_14_N_2_S		Not found in the DNP
	4.58	C_27_H_32_O_9_	Verrucarin B (**14**)	
	5.04	C_31_H_55_N_5_O_7_		Emericellamide A (**10**)
	6.61	C_18_H_32_O_2_		Linoleic acid

## 3. Discussion

Among the natural sources of bioactive compounds, microbes are more likely to support a sustainable, cost-effective production through large-scale cultivation [30]. In spite of the wide array of bioactivities found in marine and marine-derived microbes, the role of fungi from aquatic habitats—including those associated with marine organisms—as a source of antifouling substances, has been largely underestimated [31]. It was only in recent years that several antifouling compounds have been isolated from marine-derived fungi [32,33,34,35,36] and even patented as additives for antifouling coatings [37].

In this study, several aquatic ecosystems were screened for the presence of filamentous fungi (Table 1). A total of 75 isolates were recovered and identified on the basis of their microscopic and macroscopic features. Up to 21 different genera were isolated, thus representing a wide ecological diversity. This richness is particularly evident in the mangroves: 12 isolates were recovered from the sediments from *Rhizophora mangle*, whereas the rhizosphere of *Laguncularia racemosa* and *Avicennia germinans* yielded nine and seven isolates, respectively. Among the endophytes, the corals *Diploria strigosa* and *Siderastrea siderea* were particularly prolific, with five different strains being isolated from each specimen (Table 1). All the isolates were facultative and not marine obligates, that is, they are also found in terrestrial environments [38]. This does not mean that these strains are not ‘truly’ marine; indeed, they are considered as a highly specialized group of microbes of major ecological importance with particular physiological adaptations [39]. Batch cultures of the isolates reached the deceleration/stationary phase within 7 days, with biomass yields in the range 8–16 g·L^−1^ that coincide well with the values reported for other filamentous fungi isolated from aquatic environments [40] and highlight their potential for being cultured at a larger scale.

It is well known that filamentous fungi secrete secondary metabolites to the surrounding medium that are involved in diverse processes, including chemical defense towards microorganisms [41,42]. However, the secretion, or even the production of these bioactive molecules must often be triggered by or is subjected to environmental factors, for example, by a limiting nutrient availability, under different conditions of pH, temperature and/or salinity, or by inducing a competitive pressure by co-cultivation with other microorganisms [43,44]. This may explain the apparent lack of difference between the number of active extracts from the spent media and those from the fungal biomass.

Remarkably, the most active samples were identified among endophytic fungi from reef organisms. It is interesting to note that three of these isolates were obtained from corals (LAEE13, LAEE14, and LAEE21), two of them from the same species, *Diploria strigosa* (Table 1). Corals are indeed the paradigm of a holobiont, the term coined by Lynn Margulis in the 1990s to design an organism and its associated microbial symbionts. Thus, corals are hosts of a plethora of microorganisms that live in a mutualistic association [45]. Although previous studies have focused on the ability of whole-organism extracts [46,47], coral-associated bacteria [48,49,50] or bacteria associated to endosymbiotic dinoflagellates [51] to interfere with QS-regulated phenotypes, this is, to the best of our knowledge, the first evidence of this kind of behavior in marine endophytic fungi. It is tempting to hypothesize a possible ecological role of these fungal symbionts on the antifouling protection of their hosts.

The classical fungal taxonomy has been mainly based on macro-morphological and microscopical features, and this approach has not always given a reliable classification of closely related organisms. Over the last decades due to improvements in molecular techniques, this classification has been remodeled, and we have seen a revolution in many aspects of the fungal taxonomy by using genes for a molecular identification, especially functionally conserved genes as the ribosomal genes, giving a more reliable and objective classification of the organisms. The ITS regions are part of the ribosomal DNA operon. They are located between the 18S and 28S rRNA genes and divided into two segments, the ITS1 and ITS2, by the rRNA 5.8S. As part of the ribosomal operon, they are highly represented with multiple copies in the genome. However, the different ITS copies in the genome of a particular organism tend to be similar. The conserved regions of the flanking genes, 18S and 28S, facilitate the design of broad-range primers for PCR amplification across a broad range of fungal lineages and the more variable regions of the ITS allow their identification. Thus, ITS regions have been confirmed as important molecular markers for taxonomic studies and proposed as a universal barcode for fungal identification [52,53].

In this work, using a phylogenetic analysis based on the sequences of the ITS region, we have identified four marine endophytic fungal isolates as belonging to four genera: *Sarocladium* (LAEE06), *Fusarium* (LAEE13), *Epicoccum* (LAEE14) and *Khuskia* (LAEE21). One isolate, LAEE06, belonging to genus *Sarocladium*, could be assigned to the previously described species, *S. strictum* [54]. Isolate LAEE21 is likely to represent a novel species within the *Khuskia* clade, and interestingly another endophytic fungus from *Hevea brasiliensis* (*Khuskia* sp. PP96, Figure 3) could also belong to the same species [55]. However, the precise taxonomic position of the *Epicocum* isolate, LAEE14, could not be determined. Either the current data from the ITS sequences could resolve the *Fusarium* clade. Though the ITS sequences has, in general, a high resolution power to discriminate among fungal species, it is known that it shows some limitations to resolve large genera, like *Aspergillus* or *Fusarium*, which include closely related species, that form a “species complex” [56,57].

In recent years, some extra markers have been used, as the β-tubulin or the Elongation Factor-1α genes [54,57]. Thus, in order to precisely resolve the taxonomic position of some of our fungal endophytes, further analysis will be necessary using protein-coding markers.

**Figure 5:**
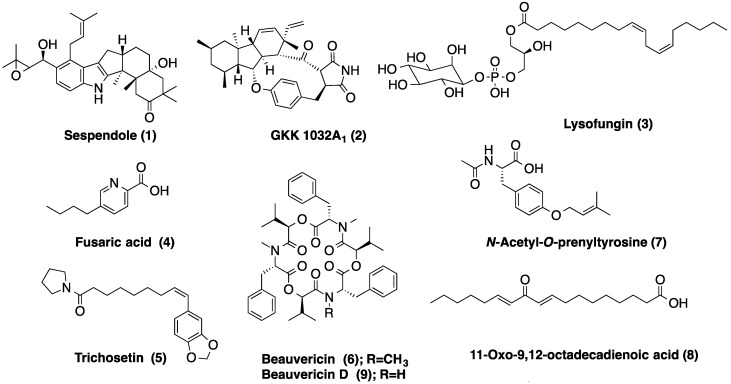

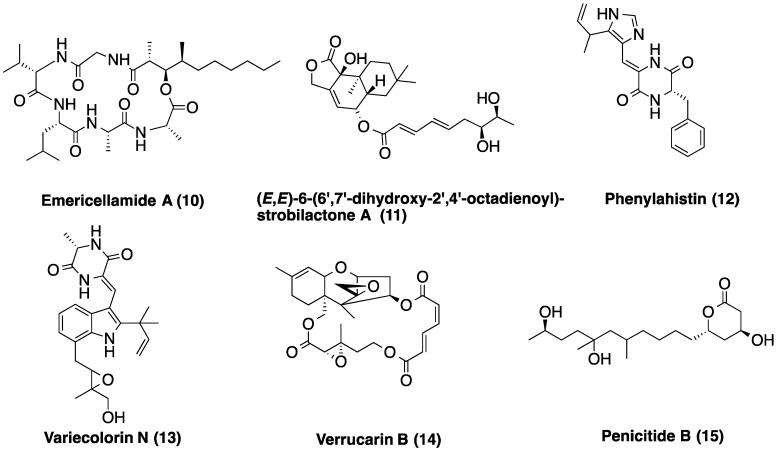
Chemical structures of representative secondary metabolites identified by LC-HRMS in the bioactive extracts.

LC-HRMS analysis of these bioactive extracts led to the identification (in-house database)/tentative identification (Dictionary of Natural Products, DNP) of several major components whose QS-inhibitory properties have not been reported so far (Figure 4 and Figure 5, Table 2). The structures of some of these main constituents are presented in Figure 5.

Strain LAEE06, extract *LAEE06C*, produced linoleic acid and three nitrogenated metabolites, two of them with the same MF and UV spectra as the previously reported fungal metabolites sespendole (**1**) and antibiotic GKK 1032A_1_ (**2**). A phosphorus-containing compound whose Molecular Formulae (MF) and UV spectra matched those of lysofungin (**3**) was also identified. The sixth molecule had a MF of C_18_H_35_NO_2_, not described in the DNP for any fungal metabolite, and therefore it may represent a new natural product.

Fusaric acid (**4**), trichosetin (**5**) and beauvericin (**6**) were identified as the main components of *LAEE13B*. Several other compounds whose MF and UV spectra were coincident with those of *N*-acetyl-*O*-prenyltyrosine (**7**), 11-oxo-9,12-octadecadienoic acid (**8**) and beauvericin D (**9**) compound with MF C_13_H_16_O_3_ and UV-Vis λ_max_ of 226; 275 nm matched five coincidences in the DNP, whereas a nitrogenated compound with MF C_9_H_13_NO and UV-Vis λ_max_ of 230(sh); 276; 301 nm did not match any previously reported fungal metabolite.

Strain LAEE14 (extract *LAEE14B*) also produced linoleic acid, emericellamide A (**10**) and (*E*,*E*)-6-(6′,7′-dihydroxy-2′,4′-octadienoyl)-strobilactone A (**11**). Two components with MF C_20_H_22_N_4_O_2_ and C_24_H_29_N_3_O_4_ were tentatively classified as two diketopiperazines (DKPs), namely phenylahistin (**12**) and variecolorin N (**13**). DKPs are not only a widely recognized group of QSIs [58] but also a family of autoinducer signals in bacterial intercellular communication [59], being able to act, consequently, as QS agonists or antagonists. A compound with MF C_15_H_20_O_3_ and UV-Vis λ_max_ of 307 nm matched more than 50 compounds in the DNP and could not be identified, whereas another compound with MF C_25_H_34_O and UV-Vis λ_max_ of 236; 300 nm did not yield any match in the DNP.

Finally, strain LAEE21 (extract *LAEE21C*) was a source of chemically diverse metabolites, including emericellamide A (**10**), compound **11**, verrucarin B (**14**) and penicitide B (**15**). Two compounds with UV-Vis λ_max_ of 210 nm and MF C_10_H_16_O_4_ and C_15_H_22_O_4_, respectively, matched several coincidences in the DNP. Interestingly, a sulfur-containing compound with MF C_17_H_14_N_2_S and UV-Vis λ_max_ of 223; 267 nm was also detected in this extract, which did not match any known natural product and could represent a novel chemical entity. Linoleic acid was also identified in this sample. Linoleic acid and other fatty acids have been previously reported as QSIs [60,61].

It is interesting to note that several metabolites highlighted in Figure 5 have recent literature precedents in marine-derived strains. Beauvericin (**6**), first isolated in *Beauveria bassiana* as an antibacterial and cytotoxic agent [62] was later identified in *Fusarium* species [63] and, last year, in a mangrove-endophytic *Aspergillus terreus* [64]. Emericellamide A (**10**) was recently isolated from the marine-derived fungus *Emericella* sp., an epiphyte of the green alga *Halimeda* sp. [65], and later from *A. nidulans* [66]. (*E*,*E*)-6-(6′,7′-dihydroxy-2′,4′-octadienoyl)-strobilactone A (**11**) has been reported in two marine-derived *Aspergillus* species: *A. ustus* and *A*. *insuetus*, isolated from sponges and algae [67,68,69]. Ocean sediments were the source of *Penicillium griseofulvum*, from which variecolorin N (**13**) was isolated for the first time [70]. Finally, the isolation of penicitide B (**15**) from *P. chrysogenum*, an endopytic strain of a *Laurencia* sp., was reported in 2011 [71].

The widespread distribution of the secondary metabolites reported in this work within different fungal genera complicates the identification of clear chemotaxonomic markers in the metabolic profiles. In spite of this fact, a clear ‘chemotaxonomic fingerprint’ was observed for LAEE13, clustered within the *Fusarium* genus (Figure 3). For this isolate, fusaric acid (**4**), trichosetin (**5**) and two beauvericins (**6**, **9**) were identified (Figure 4, Table 2). These compounds are frequent in *Fusarium* [72,73,74]. Fusaric acid is particularly renowned and, in addition, its ability to interfere with bacterial QS has been previously documented [75,76].

## 4. Experimental Section

### 4.1. Sampling and Isolation of Filamentous Fungi

A total of 75 fungal isolates from different aquatic ecosystems were recovered (Table 1). The isolation and small-scale fermentation was conducted as described by Kjer and co-workers [77] with slight modifications. In all the cases, the isolation medium was Marine Agar (Conda) supplemented with malt extract (15 g·L^−1^) and cloramphenicol (0.2 g·L^−1^), and the fermentation medium was Wickerham’s medium [77]. Details are provided below:

#### 4.1.1. Marine Endophytic Fungi

Small pieces of corals, sponges, algae and anemones were obtained by diving at a depth of 1–2 m at the Veracruz Coral Reef System National Park, Mexico, around Isla de Sacrificios (19.1750, −96.0933) and Arrecifes Blancas (19.0865, −95.9984). The samples were deposited in sterile 50 mL tubes, filled with seawater and transported to the laboratory within 2 hours. The collected samples were washed three times with sterile seawater, the external surface being disinfected with 70% ethanol. The tissues were cut into small (*ca.* 1 cm^2^) pieces aseptically. Three pieces from each specimen were placed inside a sterile tube containing 20 mL sterile seawater and homogenised with a tissue homogeniser. Dilutions 1:10, 1:100 and 1:1000 in sterile seawater were plated (1 mL) by triplicate onto agar plates. The plates were incubated at 27 ± 2 °C. Pure cultures were obtained by subsequent plating of fungal colonies.

#### 4.1.2. Root-Associated Fungi from Mangroves

Five sediment samples from each selected mangrove species (Table 1) were collected at Tampamachoco mangrove forest (21.0122, −97.3370). Samples were obtained from the area near the roots of each tree trunk using a hand auger. Only the part corresponding to the rhizosphere at an average depth of 15 cm was taken. The distance between sampling sites was 30 m on average. Sediments (10 g) were diluted with 90 mL of a sterile solution (0.1% peptone, 0.4% NaCl). Dilutions 1:10, 1:100 and 1:1000 were plated (1 mL) by triplicate. Agar plates were incubated at 27 ± 2 °C until fungal development. Fungal colonies were plated subsequently until the obtaining of pure cultures.

#### 4.1.3. Fungi from Saline Lakes

A series of samples were obtained from two saline lakes, the Alchichica Lake (19.4130, −97.4030) and the Atexcac Lake (19.3342, −97.4501), located in the state of Puebla, Mexico. The salinities of these lakes are reported in the range from 7.0 to 8.5 g·L^−1^ NaCl for Alchichica and from 6.00 to 7.0 g L^−^^1^ NaCl for Atexcac [78]. Both lakes are alkaline, with water pH values of 8.9–9.0 for Alchichica and 8.4–8.5 for Atexcac [78]. Water and sediment samples from the littoral areas of the lakes, as well as from the growing vegetation were stored in sterile flasks (water, sediments) or sterile polyethylene bags (vegetation). Diluted (1:10, 1:100, 1:1000) water samples (1 mL) were evenly distributed over the surface of an agar plate with the aid of a Drigalski loop and incubated at 27 ± 2 °C. The pH of the medium was adjusted to 8.4. Sediment samples (10 g) were diluted with a sterile saline solution (0.1% peptone, 0.4% NaCl). Dilutions 1:10, 1:100 and 1:1000 were deposited (1 mL) and streaked onto agar plates, by triplicate. Plant samples were washed first with water to eliminate the debris, then with a 4% sodium hypochlorite solution and finally with sterile distilled water. One-cm^2^ pieces were cut with a sterile razor blade and deposited onto agar plates. The plates were incubated at 27 ± 2 °C. Fungal growth was observed after 7–10 days. To identify the fungi recovered, microscopic and macroscopic features as well as taxonomic keys were used [79,80].

### 4.2. Small-Scale Fermentation and Preparation of Fungal Extracts

Once pure cultures were obtained, confirmed by macroscopic and microscopic analysis, laboratory-scale fermentation was conducted. Five hundred-milliliter Erlenmeyer flasks containing 50 mL of Wickerham’s medium were inoculated with the fungal strains. The flasks were incubated at 27 ± 2 °C under constant agitation (100 rpm) for 1–2 weeks, depending of the growth rate of each isolate. At least two replicate flasks were prepared for each fungal strain. After the incubation period, the fungal biomass (*strain code B*) and the culture medium (*strain code C*) were separated by filtration. Both samples were lyophilized and extracted with a mixture of methanol and chloroform (1:1). Subsequently, the solvent was eliminated under reduced pressure. Stock solutions (40 mg·mL^−1^) for the biological tests were prepared in microcentrifuge tubes by addition of the appropriate amount of dimethylsulfoxide (DMSO).

### 4.3. Quorum Sensing Inhibition

The reporter strain *Chromobacterium violaceum* CVO26 (CECT 5999) was used to screen the ability of fungal metabolites to interfere with violacein production, a QS-regulated phenotype. *Chromobacterium violaceum* CVO26 is a mini-Tn5 mutant that depends on an exogenous source of autoinducer (*N*-hexanoyl homoserine lactone, HHL) for violacein production. *C*. *violaceum* CVO26 was cultured in Luria-Bertani broth (Aldrich) supplemented with 25 µg·mL^−1^ kanamycin. An overnight culture was diluted 1:100, and 100 µL of this suspension were transferred to each well of a microtiter plate containing the test samples. Two batches of plates were prepared: to the first batch, 100 µL of culture medium containing the extracts (100 µg·mL^−1^) were added. These plates served as a toxicity control to evaluate the effect of the fungal extracts on bacterial growth. To the second batch, 100 µL of culture medium containing the extracts and HHL at a final concentration of 3 µM [81] were added. Both sets of plates were incubated at 28 °C with agitation (150 rpm) for 24 h.

Growth inhibition was quantified in the first batch of plates by re-suspending bacterial pellets and measuring the optical density (OD) at 625 nm in a microplate reader (Perkin-Elmer EnSpire, Waltham, MA, USA). From the second batch of plates, violacein was extracted and quantified as described by Martinelli and co-workers [82]. Briefly, the plates were dried overnight (60 °C) and violacein was resolubilized by the addition of 200 µL of DMSO. The plates were shaken for 3–4 h and then the OD_590_ was determined. Each extract was evaluated in triplicate. From the initial screening, those extracts displaying more than 40% of violacein inhibition were selected for further analysis. For these extracts, serial dilutions (500, 400, 300, 200, 100, 50 µg·mL^−1^) were prepared and assayed as described above. Each extract was evaluated by triplicate.

### 4.4. Statistics

Data were analyzed by one-way ANOVA with Dunnett’s multiple comparisons test (Sigmaplot 12, Systat Software Inc., San José, CA, USA). The assumptions of independence, normality (Saphiro–Wilk test) and homoscedasticity were verified prior to ANOVA. The limit for statistical significance was set at *p* = 0.05.

### 4.5. DNA Extraction, PCR Amplification and Sequencing

DNA was extracted from fungal cells by high-speed cell disruption [83]. Fungal hyphae were suspended in 100 µL of Tris-EDTA buffer (10 mM Tris, 1 mM EDTA, pH 8.0). Glass beads (0.5 mm diameter) were added and the mixture was vortexed for 90 s, followed by centrifugation at 15,000 rpm (3 min). The samples were finally incubated at 65 °C for at least 1 h before their use. The ITS region (ITS1, 5,8S rRNA and ITS2), was amplified using pair of primers ITS-1 (5′-TCCGTAGGTGAACCTGCGG-3′) and ITS-4 (5′-TCCTCCGCTTATTGATATGC-3′). The PCR amplification was conducted on a 25 µL reaction mixture containing 1.25 U of KapaTaq ReadyMIX DNA polymerase (KapaBiosystems), 15 pM of each primer pair and about 50 nM of the template DNA, with the temperature profile as previously described [84]. The PCR products were resolved by electrophoresis agarose gels 1% (w/v). The gels were stained with ethidium bromide for visual examination. The PCR-amplified products were purified using Quiaquick Extraction Kit (Qiagen, Venlo, Netherlands) and sequenced in an ABI3730XL sequencer (Macrogen, Inc., Amsterdam, Netherlands).

### 4.6. Phylogenetic Analysis

DNA sequences were assembled and multiple alignments performed using CLUSTAL W implemented in MEGA5 [85] software package (v.5.2.0). Phylogenetic relationships were constructed using, the Neighbour-Joining (NJ) method, and Maximum Likelihood (ML) method. The NJ tree was built using Kimura’s 2-parameter (K-2p) distance model. For ML, the Modeltest 3.7 [86] was used to select the best nucleotide substitution model, and the tree was built by using PhyML 3.0 [87]. The confidence of the branches was determined with 1000 and 500 bootstrap replications for NJ and ML, respectively. Data of the ITS sequences generated in this study are available in the EMBL database under the accession numbers: LN607808-LN607810 and LN626719, respectively for strains LAEE13, LAEE06, LAEE21 and LAEE14.

### 4.7. LC-HRMS Metabolic Profiling

For LC-HRMS, samples were analyzed using an Agilent 1200 Rapid Resolution HPLC interfaced to a Bruker maXis mass spectrometer. The volume of sample injected was 2 µL. A Zorbax SB-C8 column (2.1 × 30 mm, 3.5 µm particle size) was used for the separation. Two solvents were used as mobile phase: solvent A water:AcN 90:10, solvent B water:AcN 10:90, both with 13 mM ammonium formiate and 0.01% TFA and flow of 0.3 mL min^−1^. The gradient composition was: 0–6 min: 90% A, 10% B; 6–8.1 min: 0% A, 100% B; 8.1–10 min: 90% A, 10% B. The mass spectrometer was operated in positive electrospray ionization (ESI) mode. The instrumental parameters were: 4 kV capillary voltage, drying gas flow of 11 L·min^−1^ at 200 °C, nebulizer pressure at 2.8 bars. TFA-Na cluster ions were used for mass calibration of the instrument prior to samples injection. Each sample run was recalibrated by infusion with the same TFA-NA calibrant before the chromatographic front. Each chromatographic run was processed using Bruker algorithm for components extraction, and the most intense peaks of each run, either by UV or positive TIC were considered for interpretation of exact mass and molecular formula by studying the extracted mass components. The retention time and exact mass of the chosen components were compared against Fundación MEDINA high resolution mass spectrometry database (FM Database), and when a match was obtained it was reported as a named compound. For the components with no matches in the FM Database, the obtained molecular formula/exact mass was searched against the Chapman and Hall Dictionary of Natural Products database. If a plausible match was found—considering the exact mass/molecular formula, the producing microorganism and the target assay—the molecule was reported as a suggested component of the fraction.

For LC-LRMS, the chromatographic system is an Agilent 1100 and the mass spectrometer is a single quadrupole Agilent MSD. The solvents and chromatographic gradient are identical in both systems, however, due to the dead volume optimization performed by Agilent in the new 1200 RR model, elution in the LC-HRMS tends to be earlier than in the LC-LRMS. The mass spectrometer was operated in positive and negative ESI mode (scan). The instrumental parameters were: 3.5 kV capillary voltage, drying gas flow of 11 L·min^−1^ at 325 °C, nebulizer pressure at 40 psig. Database matching was performed using an in-house developed application where the DAD, retention time, POS and NEG mass spectra of the active samples were compared to the UV-LC-MS data of known metabolites stored in a proprietary database where metabolite standard data were obtained using the exact same LC-MS conditions as the samples under analysis.

## 5. Conclusions

This study highlights the potential of fungal secondary metabolites to interfere with bacterial QS. Although fungi are a renowned source of bioactive compounds, their ability to thwart bacterial cell-to-cell communication is poorly characterized. Among the diversity of aquatic environments screened in this study, marine endophytes clearly stood out. With the exception of *LAEE13B*, the QS-disrupting activity exhibited by the extracts of these endophytic filamentous fungi was unrelated to toxic effects on the reporter strain *Chromobacterium violaceum* CVO26. LC-HRMS profiles of these extracts revealed the presence of a broad diversity of fungal metabolites (Figure 5), some of them of recent identification in marine-derived strains [62,63,64,65,66,67,68,69,70,71]. Interestingly, all these fungal extracts contained compounds that might represent new natural products whose identity and biological activity will be determined in further experiments. The identity of the compounds tentatively identified by HRMS and UV analysis and whether the presence of the abovementioned metabolites can explain the QS-inhibitory activity observed in the extracts or it is due to the presence of minor components, or even to the synergistic effect of combinations of these molecules are issues that remain to be determined. To the best of our knowledge, this is also the first report of QS inhibition in endophytic fungi isolated from marine organisms.

## Supplementary Files

Supplementary File 1
