# Supplementary material for: Inhibition of Bacterial Quorum Sensing by Extracts from Aquatic Fungi: First Report from Marine Endophytes
Source: Mar Drugs. 2014 Nov 19;12(11):5503–26. doi: 10.3390/md12115503 (PMC4245542; doi:10.3390/md12115503)
Supplement: Supplementary File 1 [file marinedrugs-12-05503-s001.pdf]

Supplementary Information

Figure S1. Neighbour-joining (Kimura-2 parameter model) phylogenetic tree based on ITS sequences (552 nt) of isolates of this study and strains belonging to culture-collection. Accession numbers are given in parentheses. Numbers at the nodes are bootstrap support values (≥50%) for 1000 replicates. The scale bar indicates the number of substitutions per site.

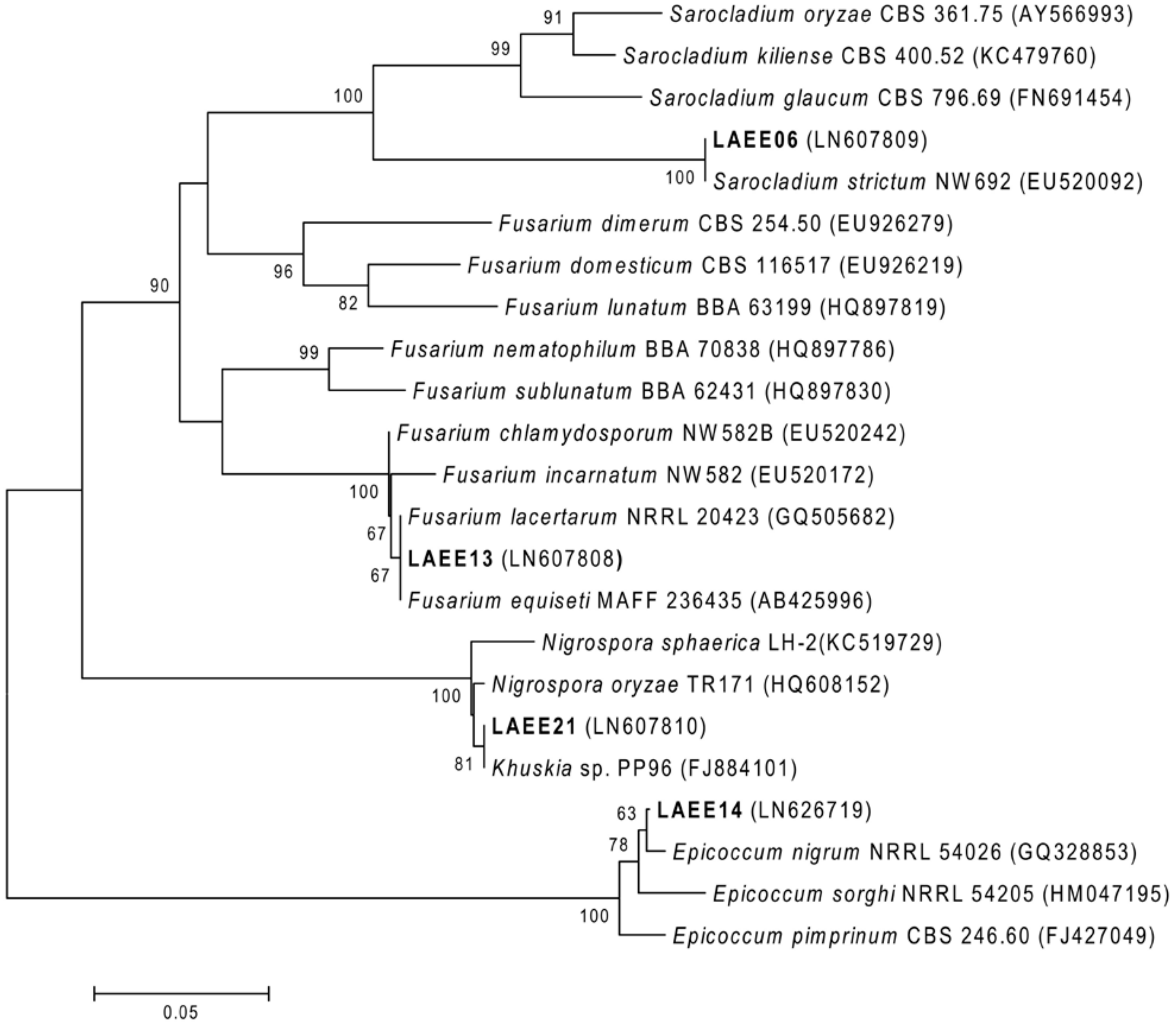

© 2014 by the authors; licensee MDPI, Basel, Switzerland. This article is an open access article distributed under the terms and conditions of the Creative Commons Attribution license (http://creativecommons.org/licenses/by/4.0/).
